# Environmental drivers of dynamic soil erosion change in a Mediterranean fluvial landscape

**DOI:** 10.1371/journal.pone.0262132

**Published:** 2022-01-21

**Authors:** Nazzareno Diodato, Francesco Fiorillo, Massimo Rinaldi, Gianni Bellocchi

**Affiliations:** 1 Met European Research Observatory, International Affiliates Program of the University Corporation for Atmospheric Research, Benevento, Italy; 2 Department of Science and Technologies, University of Sannio, Benevento, Italy; 3 Department of Earth Sciences, University of Florence, Florence, Italy; 4 Université Clermont Auvergne, VetAgro Sup, INRAE, Clermont-Ferrand, France; Jinan University, CHINA

## Abstract

**Background:**

Rainfall and other climatic agents are the main triggers of soil erosion in the Mediterranean region, where they have the potential to increase discharge and sediment transport and cause long-term changes in the river system. For the Magra River Basin (MRB), located in the upper Tyrrhenian coast of Italy, we estimated changes in net erosion as a function of the geographical characteristics of the basin, the seasonal distribution of precipitation, and the vegetation cover.

**Methods and findings:**

Based on rainfall erosivity and surface flow and transport sub-models, we developed a simplified model to assess basin-wide sediment yields on a monthly basis by upscaling the point rainfall input. Our calibration dataset of monthly data (Mg km^-2^ month^-1^, available for the years 1961 and 1963–1969) revealed that our model satisfactorily reproduces the net soil erosion in the study area (*R*^*2*^ = 0.81). For the period 1950–2020, the reconstruction of an annually aggregated time-series of monthly net erosion data (297 Mg km^-2^ yr^-1^ on average) indicated a moderate decline in sediment yield after 1999. This is part of a long-term downward trend, which highlights the role played by land-use changes and reforestation of the mountainous areas of the basin.

**Conclusion:**

This study shows the environmental history and dynamics of the basin, and thus the varying sensitivity of hydrological processes and their perturbations. Relying on a few climatic variables as reported from a single representative basin location, it provides an interpretation of empirically determined factors that shape active erosional landscapes. In particular, we showed that the most recent extreme storms associated with sediment yield have been characterised by lower cumulative rainfall, indicating a greater propensity for the basin to produce sediment more discontinuously over time.

## Introduction

Understanding the long-term effects of hydrological variability and extremes [1–3] on the variability of soil erosion and sediment delivery is a challenge for environmental science [4]. This is because individual rainfall events, whose magnitude and frequency affect soil erosion, are nested in longer-term patterns of environmental change [5]. In Italy, located in the centre of the Mediterranean region, where extreme rainfall events often appear elusive or in the form of small erratic clusters [6], agronomists and travellers have documented the timing of damaging hydrological phenomena and soil erosion already in historical times [7]. Suffice it to recall the Italian Renaissance polymath Leonardo da Vinci (1452–1510), who denounced the dangers of a degradation of the hilly and mountainous landscapes, already underway in various parts of the Italian peninsula and in particular in his region, Tuscany [8, p. 201]:

“«*Li monti–aveva avvertito Leonardo da Vinci–sono disfacti dalle piogge e dalli fiumi»*, *e già Pietro de’ Crescenzi*, *qualche secolo prima*, *aveva raccomandato la lavorazione di traverso dei terreni collinari*, *altrimenti la terra “sarebbe tutta portata via dalla pioggia alla valle quad’ella discende con empito dalla pendice del monte*”

[The mountains–Leonardo da Vinci had warned–are devastated by rains and rivers», and already Pietro de’ Crescenzi, a few centuries earlier, had recommended working across the hilly land, otherwise the land “would all be carried downstream when it descends strength from the slopes of the mountains”]

Even after this period and up to present day, most Tuscan river basins have been strongly influenced by various types of climatic forcings and human disturbances, mainly from land-use changes related to agriculture, flood mitigation, and exploitation of natural resources [8–10]. Hillslope processes and channel adjustments can cause a series of problems (e.g. damage to built-up areas, infrastructure, loss of soil, possible flooding linked to sedimentation processes), although in many cases they can also have beneficial effects for ecosystems, spontaneously promoting habitat diversification [11]. Thus, knowledge of the evolutionary trends of these basins is essential to ensure the protection and safety of rivers, as well as their management and restoration.

While great advances have been made in the understanding, description and modelling of sediment discharge and soil erosion [12], little attention has been paid to long-term modelling because monitoring of hydrological and erosion processes is only available for short periods [13]. Satellite data also do not allow accurate detection of monthly or annual variations in sediment production [14]. Follow-up modelling and future developments could unlock this limitation and resolve important variations in sediment production at different time-scales. Physics-based numerical approaches can complement soil erosion experimentation [15] but may be unwieldy due to problems of identifiability and data availability [16]. Indeed, despite considerable advances in model-based responses to sediment erosion, experimental research on erosion in the context of long-term environmental and climatic changes continues [17].

In Mediterranean environments, conceptually sound, regression-based models can provide a simple and parsimonious interpretation of the erosion response of homogeneous basin units and can be more easily adopted over longer time-scales [18]. In particular, they can capture the effects that climate have had on long-term (e.g. decadal scale) historical fluctuations in hydrological extremes [19], while also capturing local flash-flood events occurring in Mediterranean basins [20]. The occurrence of these events has increased in recent times, resulting in dramatic mass movements and sediment discharges [21]. Alongside these events, a limited number of works have focused on the long-term annual estimation of rainfall erosivity in the Tuscan-Ligurian sector [22], leaving the Magra River Basin (MRB) free from investigations on soil erosion in specific months or years. Also in contrast to many studies conducted on individual extreme hydrological events [23, 24], and their geomorphological [25] and geological [26] effects, along the MRB there is a paucity of long-term research on soil erosion. Exceptions are the works of Rinaldi et al. [11, 27], who analysed the spatial pattern of channel changes along the Magra River providing possible options for sediment management.

Although rainfall and sediment discharge have been historically monitored in Italy, erosion measurements have not been prolonged over the last decades, limiting the development of hydrological models and their application to understand the effect of soil erosion drivers [28]. For this reason, we applied an approach to sediment assessment incorporating basin-scale knowledge and management of sediments at the basin scale, and a broader application of available knowledge in hydroclimatology and geomorphology. With focus on the MRB, we developed a monthly-based parsimonious erosion model based on concepts of Foster et al. [29] and Thornes [30] for which soil erosion can be simulated based on runoff, slope gradient, vegetation cover and soil erodibility. Our integrative methodology incorporates experimental hydrological data into a regression-derived erosion model that upscales the input data from point rainfall to the basin area where hydrological processes respond. This resulted in a sufficiently long time-series of single-station rainfall input data (1950–2020) for the parsimonious model, whose evaluation in the MRB offered a unique opportunity to explore geomorphological processes in this fluvial basin. We refer here to net soil erosion (Mg km^-2^), i.e. the mean sediment yield (or production) that occurs throughout the basin over time, resulting from the sum of sediment produced by all sources of erosion, including surface flow, ephemeral gullies and stream channel areas.

The objective was to capture the multiple factors of a changing environment (including climate, vegetation cover and erosive-resistance changes) with readily available data. In doing so, we sought to present an overview of the trend in soil erosion over the time period considered together with the main factors that have driven this trend in a Mediterranean river landscape.

## Materials and methods

### Study area

**Fig 1:**
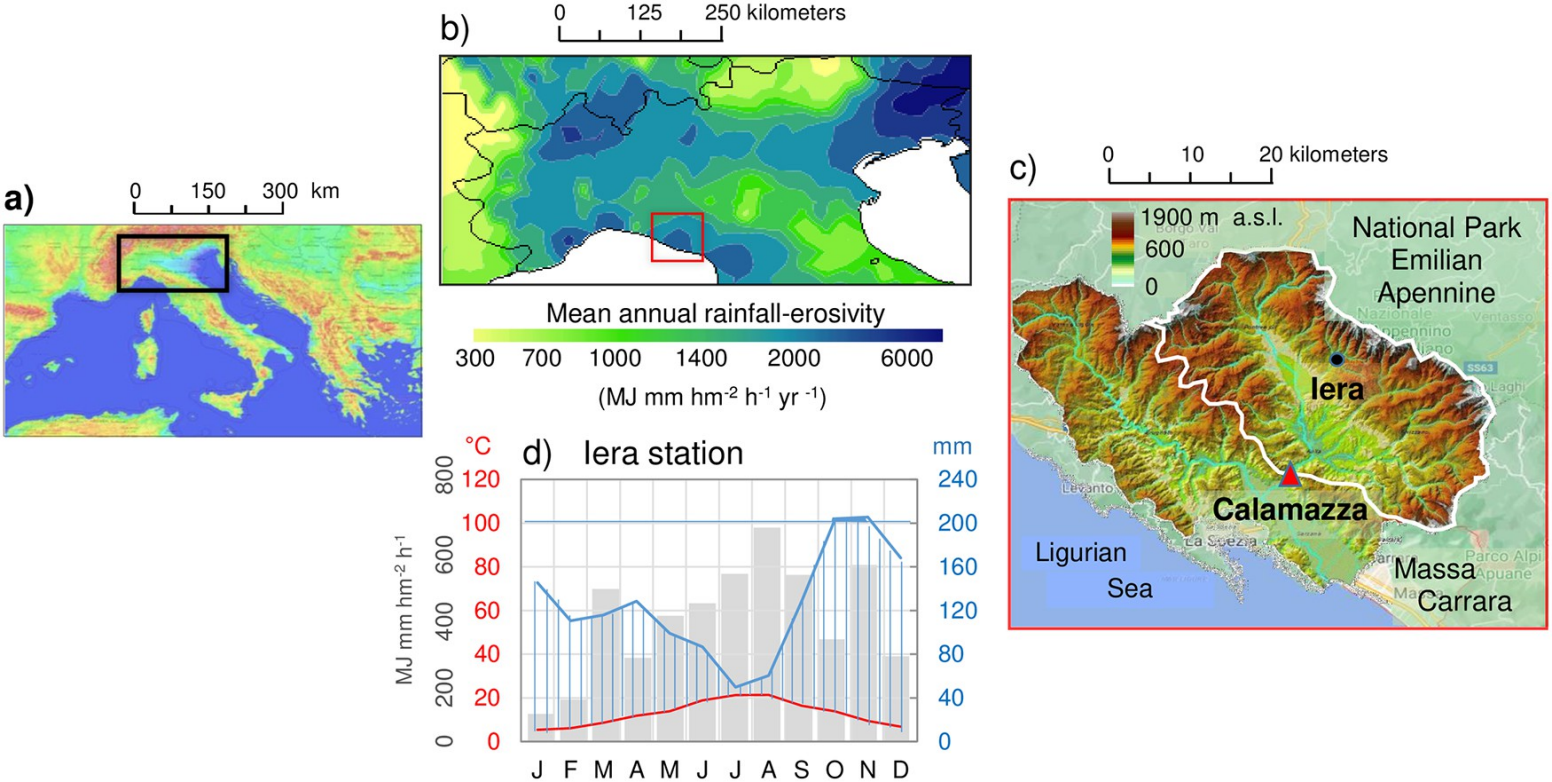
Environmental framework of the Magra River Basin. a) northern sector of Italy in the central Mediterranean area, b) rainfall erosivity map of northern Italy [31] with the study area (red square), c) digital elevation model of the basin with the basin outlet at the hydrometric station of Calamazza (red triangle) and the reference pluviometric station of Iera (black dot), d) monthly rainfall erosivity (grey bars, MJ mm hm^-2^ h^-1^ month^-1^), and Walter-Lieth diagram showing the monthly regime of air temperatures (red curve) and precipitation (blue curve, with blue areas in February and November indicating that precipitation exceeds 200 mm in these months). Maps are authors’ own elaboration from free, public domain, images: a) extraction from Geological Survey of Italy Portal (http://sgi2.isprambiente.it/viewersgi2); b) analysis from ArcGIS-ESRI Geostatistical Analyst on the ESDAC (European Soil Data Centre) dataset (https://esdac.jrc.ec.europa.eu/content/global-rainfall-erosivity) and c) adaptation from Magra River Basin Authority [32]”.

Centred around 44° 17′N and 09° 57′E, Val di Magra (Magra valley) is a characteristic area on the western Italian side, between northern and central Italy, enclosed by the Parma Apennines, the Apuan Alps and the Ligurian Apennines (Fig 1). The Magra River Basin (MRB) has an extension of ~1700 km^2^ and consists of a system of two parallel valleys: to the west the valley of the river Vara, its main tributary, and to the east the upper middle valley of the river Magra. For the purpose of this study, only the Magra basin near the outlet of the Calamazza (44° 12’, 09° 57’, 45 m a.s.l.) hydrometric station (the easterly part with a surface area of 939 km^2^) was considered (Fig 1c). The locality of Iera (44° 19’ N, 10° 02’ E, 547 m a.s.l.) was the reference station for the rainfall data input to the hydrological model.

**Fig 2:**
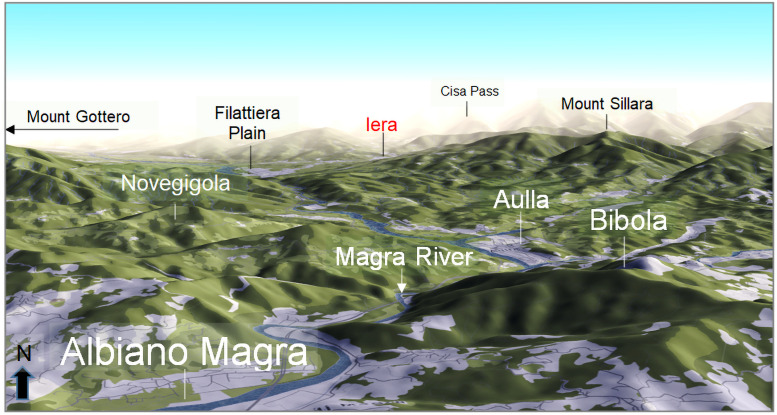
Perspective view of the Magra landscape. The Magra River crosses the valley from north to south passing through the borough of Novegigola (commune of Tresana, 44° 14′ N, 09° 57′ E), and the city of Aulla (44° 13′ N, 09° 58′ E) and its fractions Albiano Magro and Bibola. The site of Iera (commune of Bagnone, 44° 18′ N, 09° 59′ E), which is the reference rainfall station for this study, is marked in red. The highest elevations are also indicated. Map is an output image created from OpenStreetMap (https://demo.f4map.com/#camera.theta=0.9).

The basin has a mean altitude of 612 m a.s.l., with a peak of 1904 m a.s.l. at Mount Alto (44° 19′ N, 10° 12′ E), and a mean slope of 13%. Lithologically, the MRB developed mainly on sandy and clayey-marly soils, to which calcareous and metamorphic-magmatic complexes were added (northwest side of the Apuan Alps), and with a low drainage density [33]. The related hydrographic networks show a convergent pattern in the most upstream part, probably linked to steep slopes [34], and then flow with an almost straight course oriented in the North-South direction (Fig 2). The forest area is ~75% while the arable area is ~16% [35]. The Magra River and its tributaries provide abundant water resources and a varied landscape that rises from the alluvial plain towards the hills (Fig 2), lending itself to horticulture, viticulture and olive growing [36]. In the valley bottom of the Magra River, simple arable land dominates, with riparian tree and shrub formations (willow, poplar and alder). On the mountain slopes, forest ecosystems are the dominant feature, with a predominance of deciduous forests of beech, chestnut, oak, fir and mixed conifer and broadleaved forests.

Climatologically, Val di Magra is interesting for its extreme hydrological events, which even the medieval Italian poet Dante Alighieri (c. 1265–1321) mentioned in his Divine Comedy (Inferno Canto XXIV, lines 144–146):

“*vapor di Val di Magra ch’è di torbidi nuvoli involuto; e con tempesta impetüosa e agra*”

["a bolt from Val di Magra, engulfed by torn and threatening clouds; and with violent and stinging storms"]

The reliefs of the Apennines favour the development of rainfall on the slopes and the air temperature is affected by the influence of the Tyrrhenian Sea [37]. The Walter-Lieth climatic diagram [38], which illustrates the changes in monthly precipitation and temperature at the reference station of Iera, shows that no dry month is observed during the year, i.e. the precipitation curve, in mm, is always above the air temperature curve, in °C (Fig 1d). This is in agreement with Rapetti and Vittorini [37], who showed no arid month for the whole basin and that soil water is not a limiting factor for plant activity. Proximal to the sea, the Apennine ridge that surrounds the basin to the north and east causes a forced upwelling of air and abundant rainfall over the MRB (1700 mm yr^-1^), even reaching 3000 mm yr^-1^ on the basin ridges [27]. Consequently, the mean annual rainfall erosivity is also high, with values of about 5000 MJ mm hm^-2^ h^-1^ [31]. From the above context, it is clear that much of Tuscany has always been exposed to aggressive rainfall, and the MRB is even more exposed than other areas (Fig 1b). As can be seen from Fig 1d (grey bars), montly erosivity is characterized by a marked seasonal oscillation with maximum values between summer and early autumn, when it can reach 400–600 MJ mm hm^-2^ h^-1^ [39].

### Data sources

For rainfall and net erosion, we have referred to the data provided by the Annals Project (https://www.isprambiente.gov.it), available through the ISPRA (*Istituto Superiore per la Protezione e la Ricerca Ambientale*) portal and updated by the regional administrations (until 2020 for Tuscany, https://www.sir.toscana.it/consistenza-rete). For the MRB, net erosion data are available for the years 1961 and 1963–1969, which was used for the calibration of the erosion model. The reference rainfall station of Iera, centrally locate in the MRB, was used for model calibration and reconstruction of net erosion for the period 1950–2020. Until 1980, the monthly vegetation cover percentage was calibrated annually on a constant basis, with monthly variation from January to December around the annual mean value of ~75% [35]. Subsequently (until 2020), variable values were obtained based on actual NDVI (Normalized Difference Vegetation Index) data from NOAA Climate Data Record [40].

### Model development

Sediment yield, also known as net erosion, is the sum of sediment produced by all sources of soil erosion, including those from splash erosion, overland flow and runoff, and stream channel areas [41] minus the amount of sediment deposited on these areas and in valley floodplains, which crossing the outlet of a river basin. The result is the amount of sediment transported downstream to the basin outlet, as determined by four interacting factors: climate, soil, topography, and land-use. These soil erosion drivers are taken into account in a hierarchical structure to represent the erosion phenomenon. In order to generalise the expression and take into account the role of weathering in erosion, a precipitation index is considered, which is appropriate in temperate climates with a predominance of water-driven erosion. The erosive action of rainfall depends not only on the amount of precipitation, but also on its seasonal distribution due to its concurrence with different seasonal conditions of soil erodibility, vegetation cover and agricultural practices, and, finally, on the intensity of rainfall events. Then, we assumed that hydraulically rough, plant-covered surfaces reduce the flow velocity and thus the interril transport capacity of the soil.

We developed a model concept, translated into semi-empirical REgression-Derived Erosion Model–REDEM_(MRB_)—on a monthly basis (Mg km^-2^ month^-1^), as follows:

$${REDEM}_{\left(MRB\right)}=A∙\left[\left(R_{S}+R_{Q}\right)∙e^{-0.07∙VCP}\right]∙SDR$$ (1)

where: *A* is a scale parameter to convert the bracketed term in Mg km^-2^ month^-1^, which depends on the geographical characteristics of the basin; *R*_*S*_ is the rainfall-erosivity indicator associated more with splash erosion and rill erosion, *R*_*Q*_ is the erosivity indicator associated more with surface flow and transport erosion; *e*^(-0.07 *VCP*)^ is the exponential vegetation cover function of Thornes [30], which reduces raindrop erosivity for detachment and interril sediment-transport capacity (where *VCP* is the % vegetation cover); *SDR* is the soil delivery ratio, i.e. the fraction of the gross erosion which is expected to be delivered to the outlet of the basin.

The component *R*_*s*_ was derived from Diodato and Aronica [42]:

$$R_{S}=\left(1+{dx}^{\vartheta }\right)∙f(rh)$$ (2)

where: *dx* is the maximum daily rainfall (mm) in each *j* month; $f(rh)=1-\Omega ∙cos\left(6.28\frac{j-\sigma }{\varphi -j}\right)$ is a scale-facor modulating the hourly intensity of the intra-seasonal precipitation ϑ, Ω, σ and φ are calibrated parameters. In fact, the combined effect of rainfall intensity and duration increases sediment yield (by ~24% according to Shojaei et al. [43]), compared to the single most important factor (i.e. rainfall duration).

The indicator of erosivity *R*_*Q*_ associated more with runoff erosion, was developed as follows:

$$R_{Q}=p_{m}^{\left(B_{sw}+S_{W}\right)}$$ (3)

where *p*_*m*_ is the amount of rainfall (mm month^-1^) in month *m* (1, …, 12); the exponent is an indicator of soil humidity, composed of the binary factor *B*_*SW*_, equal to 1 when *p*>200 mm and *dx*>90 mm simultaneously between July and December, and equal to 0 in all other cases, and by a semi-parametric soil humidity function (*Sw*), to modulate the intra-seasonal moisture after precipitation:

$$S_{W}=\left(\alpha +\beta ∙cos\left(6.28\frac{j-\nu }{\eta -j}\right)\right)$$ (4)

Eq (4) relies on the fact that changes in the underlying surface conditions are significant contributors to runoff and sediment yield processes [44]. This is also of great significance for further summarizing the relationships between runoff and sediment yield, and for understanding the variation of sediment yield associated with different rain events [45].

The term *SDR*, which converts gross erosion into net erosion, was estimated based on Arnold et al. [46]:

$$SDR={\left[\psi ∙\left(0.78+0.22∙\frac{\sqrt{R_{Q}}}{\left(1+dx\right)}\right)\right]}^{0.56}$$ (5)

The concept is an analogue to the connectivity ratio (the amount of sediment reaching a stream over the amount of sediment eroded), which characterises the efficiency of slope-channel transfer and depends on the transport capacity and the shape of the slope and the drainage pattern [47].

### Model assessment

The model parameterization has been derived from physical considerations, which have been successively integrated with empirical correlations with observed data. The upscaling issue formulated using Thornes [30] exponential vegetation function was also included to ensure that the mathematical formulation of a particular process that is valid at a point-station is in some way representative over an area as large as a homogeneous basin unit. The modelling procedure has been carried out iteratively over the period for which sediment data were available (1961 and 1963–1969) until a significant relationship between actual and predicted data was obtained and the following criteria were met:

$$\left\{\begin{array}{c} R^{2}=max\\ MAE=min\\ \left|b-1\right|=0 \end{array}\right.$$ (6)

i.e. maximizing the goodness-of-fit (*R*^*2*^, optimum 1) minimizing mean absolute error (*MAE*, optimum 0) and minimizing the difference from the unity of the regression slope (*b*, optimum 1) between modelled and actual data.

Spreadsheet-based model development was performed using the free online statistical software STATGRAPHIC (http://www.statpoint.net/default.aspx), with graphical support from WESSA (https://www.wessa.net/tsa.wasp) and CurveExpert Professional 1.6 (https://www.curveexpert.net).

The full set of raw data and the equations that support the findings of this study are available in S1 File.

## Results

### Model parameterization and evaluation

The criteria of Eq (6) were matched with the following calibrated parameters: *A* = 3.55, *VCP* (January, …, December): 65, 65, 70, 70, 75, 75, 77, 80, 75, 75, 70 and 70% (before 1980, estimated from NDVI data afterward: *VCP* = 100∙(0.2771∙NDVI+0.5929), r = 0.91); ϑ = 2, Ω = 0.55, σ = 6.0 and φ = 20 in Eq (2); α = 0.9, β = 0.48, ν = 3 and η = 25 in Eq (4); ψ = 0.10 in Eq (5). A highly significant regression (p~0.00) was obtained between actual and predicted data, with the *R*^2^-statistic indicating that the REDEM_(MRB)_ explains 81% of net erosion variability. The mean absolute error (*MAE*), used to quantify the amount of error, was equal to 18 Mg km^-2^ month^-1^, which is lower that the standard deviation of the residuals (27 Mg km^-2^ month^-1^).

**Fig 3:**
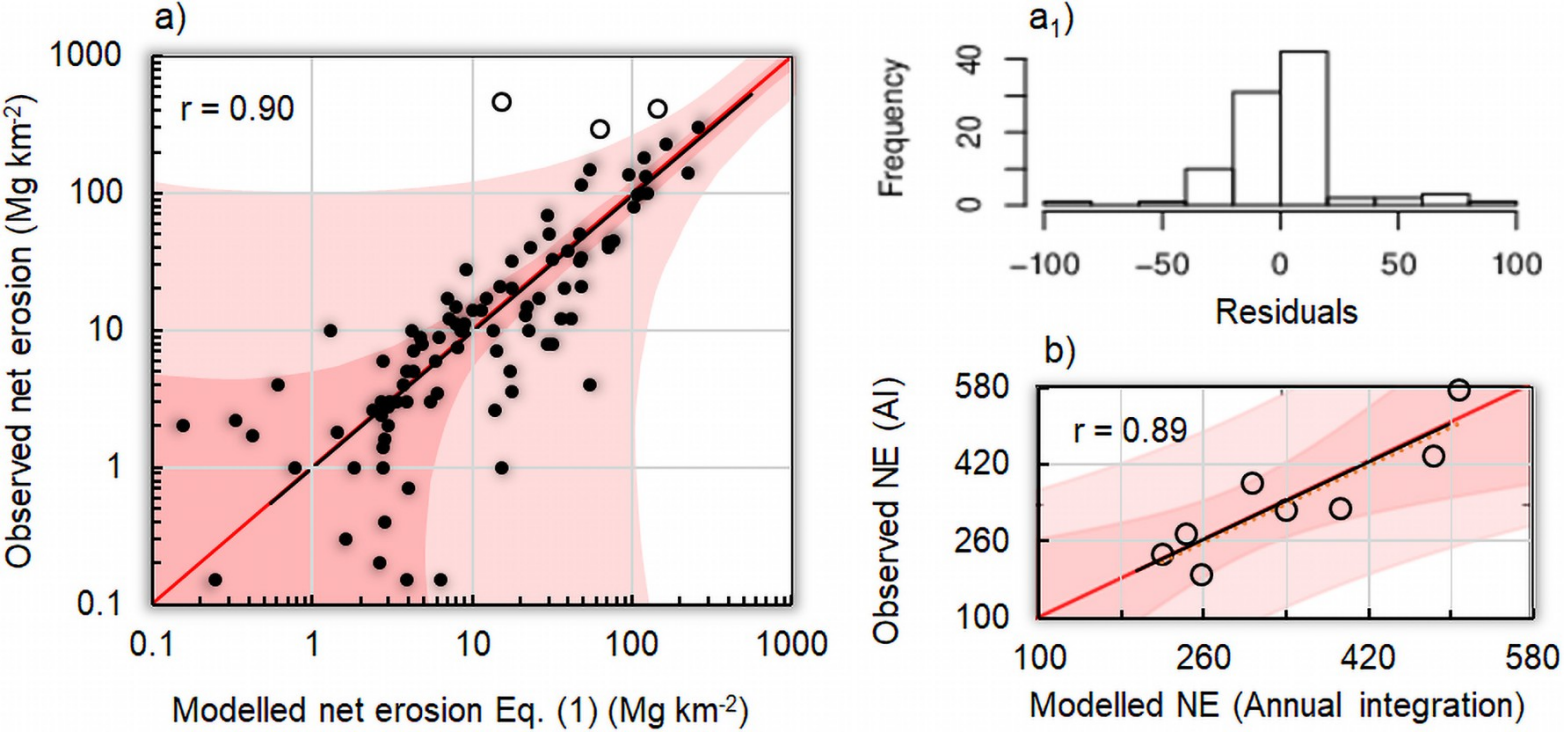
Model evaluation. a) scatterplot (log-scales) of regression model (black line and 1:1 red line) versus experimental net erosion estimated at the outlet of Magra River Basin over the years 1961, and 1963–1969 with the inner bounds showing 90% confidence limits (power pink coloured area), and the outer bounds showing 95% prediction limits for new observations (light pink), out of which three outlier data are marked in empty circle and not considered for model calibration. a_1_) residuals between actual and modelled net erosion. b): Scatterplot between the annual actual and modelled net erosion values (Mg km^-2^ yr^-1^), with 1:1 (red line), regression line (black line), and related confidence limits (power and light pink bands as in a).

Fig 3a reports the calibration results of the regression model (black line) for 96 sediment yield data-points (i.e. one determination per month). Three data (November and December 1966, and November 1967) falling outside the outer bounds showing 95% prediction limits for new observations were not considered for calibration, i.e. ~3% of the total database.

Negligible departures of the data-points from the 1:1 red line are observed (log-scales in both axes), and there is a statistically significant relationship between observed and predicted data (p<0.05). Fig 3a_1_ shows a skew-free distribution of model residuals compatible with a Gaussian pattern. The Durbin-Watson statistics (DW = 1.73, p = 0.86) shows that there is no indication of serial autocorrelation in the model residuals.

In order to evaluate the temporal invariance of the model, we aggregated net erosion on an annual basis. Year-to-year fluctuations in actual net erosion values were largely reproduced by the time-integrated REDEM_(MRB)_ for all available years (Fig 3b). Satisfactory evaluation statistics (*R*^*2*^ = 0.79 and *MAE* = 44 Mg km^-2^ yr^-1^) indicate the suitability of temporal integration for model-based interannual assessments.

### Model components and input/output relations

**Fig 4:**
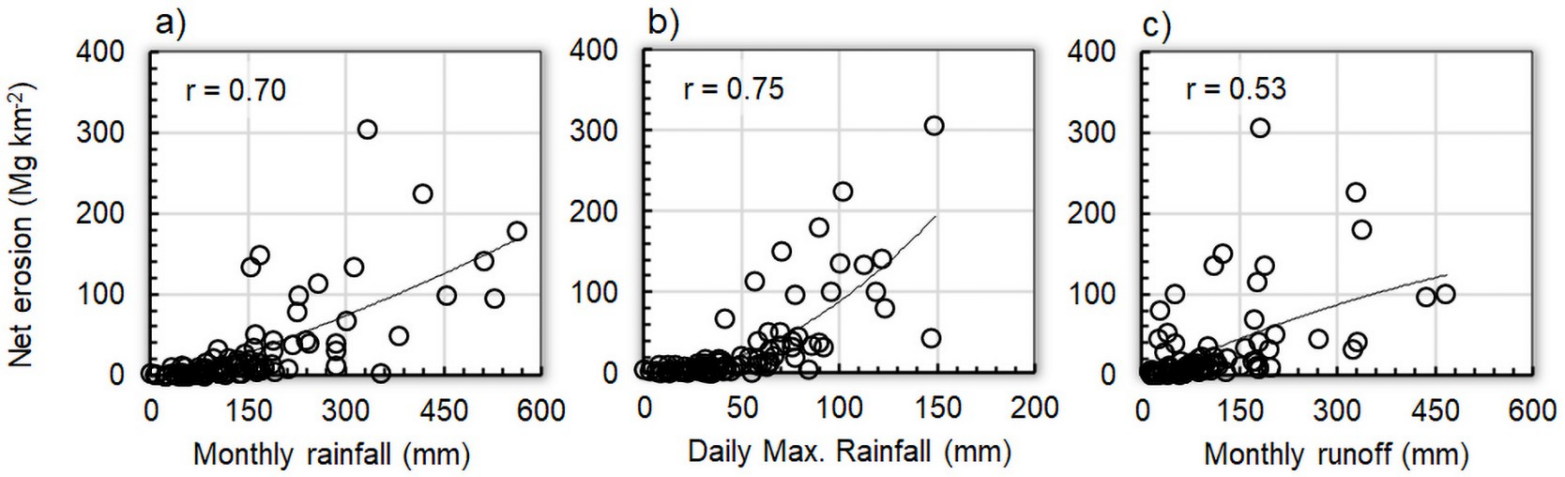
Polynomial regression modelling of monthly monthly net erosion as a function of alternative inputs. a) monthly amount of rainfall (Y = 1.28E-04·X^2^+2.49E-01·X-1.26E+01), b) daily maximum rainfall in each month (Y = 8.37E-03·X^2^+7.04E-02·X-2.63E+00), c) monthly runoff (Y = -1.91E-04·X^2^+3.67E-01·X-5.80E+00).

We adopted a 2^nd^-order polynomial, *Z* = *A*·X*+B*·X^2^+C, to assess the role played by individual estimators, where *Z* represents the monthly net erosion and X represents, alternatively, the monthly amount of rainfall (*p*), the maximum daily rainfall in a month (*dx*), or monthly runoff (*Q*). Fig 4 shows that overall net erosion increases with increasing valued of *p* (Fig 4a), *dx* (Fig 4b) and *Q* (Fig 4c). In the case of *dx* (Fig 4b), the increases of net erosion are relatively low until about 50 mm month^-1^, and then the sediment increases at a faster rate. This is reflected in the exponent ϑ = 2 in Eq (2), which amplifies the effect of high values of *dx*. With correlation coefficients between 0.53 (X = *Q*) and 0.75 (X = *dx*), runoff and maximum daily rainfall in a month (taken individually) are identified as the best and worst indicator of sediment rate, respectively.

The development of REDEM_(MRB)_—Eq (1)—is the result of a combination of such hydrological inputs, together with the role of vegetation cover. To determine whether REDEM_(MRB)_ can be simplified, we fitted a multiple linear regression model to describe the relationship between net erosion and the three independent components *R*s, *R*_Q_ and *VCP* in Eq (1). The highest p-value on the independent variables, 0.04, belonging to *VCP*, indicates that also this term is statistically significant. Consequently, Eq (1) cannot be simplified, resulting in a stable, interpretable and usable model according to Royston and Sauerbrei [48].

### Temporal and spatial scaling

In relatively small and mountainous basins, geomorphological processes are characterized by nonlinear interactions between climatic constraints, land surface and fluvial responses on different spatial and temporal scales [49]. For instance, the dynamics of sediment transport are nonlinear, as they can be strongly influenced by increased sediment availability after extreme events, such as downpours and floods, and by complex dynamics of activating sediment sources with different degrees of connectivity with drainage at the basin scale [50]. One possible approach to account for time complexity in hydrological models of river basins is to integrate fast-moving processes, such as the partitioning of rainfall into rain-splash and runoff. Hydrological models are sensitive to the time-step of the simulation, and then, in the representation of rainfall erosivity, only one indicator of the maximum hourly rainfall per month—Eq (2)–is taken, in order to limit the errors that can accumulate when aggregating intensity calculations in smaller steps. In this way, Eq (2) is adequate to account for the large variability, from month to month, of the amount of energy released between 1 and 24 hours.

As Mulligan and Wainwright [51] pointed out, the hydrological processes illustrated above are strongly dominated by the spatial connectivity of runoff-producing elements. One attempt to incorporate upscaling in our model has been to account for both the vegetation cover percentage (*VCP*), in the exponent of Eq (1), and the sediment delivery ratio (*SDR*) in Eq (5). The latter is the fraction of the eroded soil, generated by each source of the basin, that reaches the nearest permanent drainage line. Our estimate of the basin-wide *SDR* scaling factor—Eq (5)—was equal to 0.26 on an annual basis. This value, close to the value of 0.20 obtained with a detailed hydrological model in the mountainous Bilancino river basin, north of Florence [52], can be considered a reliable estimate for the quantification of the sediment delivery in the MRB, whose landscape is characterised by a mosaic of natural elements such as woods, hedges and meadows. Such a low value is also compatible with the results of Surian et al. [53], who suggested that a redistribution of stored material on floodplains was likely the dominant process during floods through the MRB.

## Discussion

### Historical development of the Magra River Basin

To discuss the effects of sedimentation processes in relation to the estimated past changes, it is useful to summarize the historical evolution of the Magra landscape up to its present conditions in order to find out from which agricultural and geomorphological context the basin originates. Disastrous hydro-geomorphological events affected the Magra valley in the past, such as the one in 1509, which submerged the valley under a thick layer of sediment ([54], p. 324):

*Accadde una rovinosa inondazione del Fiume Magra, ingrossato straordinariamente per le copiose ed incessanti piogge, narrando l’Annalista Villani, che la Magra giunse a correre per la strada della Nonziata e devastò li Forni di San Leonardo de’ Maraffi, e quello de’ Trincadini; e restarono sepolti sotto terra i Ponti del Fiume Verde*

[There was a disastrous flooding of the Magra River, which had become extraordinarily swollen by the copious and incessant rainfall, the annalist Villani recounting that the Magra reached the Nonziata road and devastated the Forni di San Leonardo de’ Maraffi, and that of Trincadini; and the Bridges of the Green River were buried under the ground]

**Fig 5:**
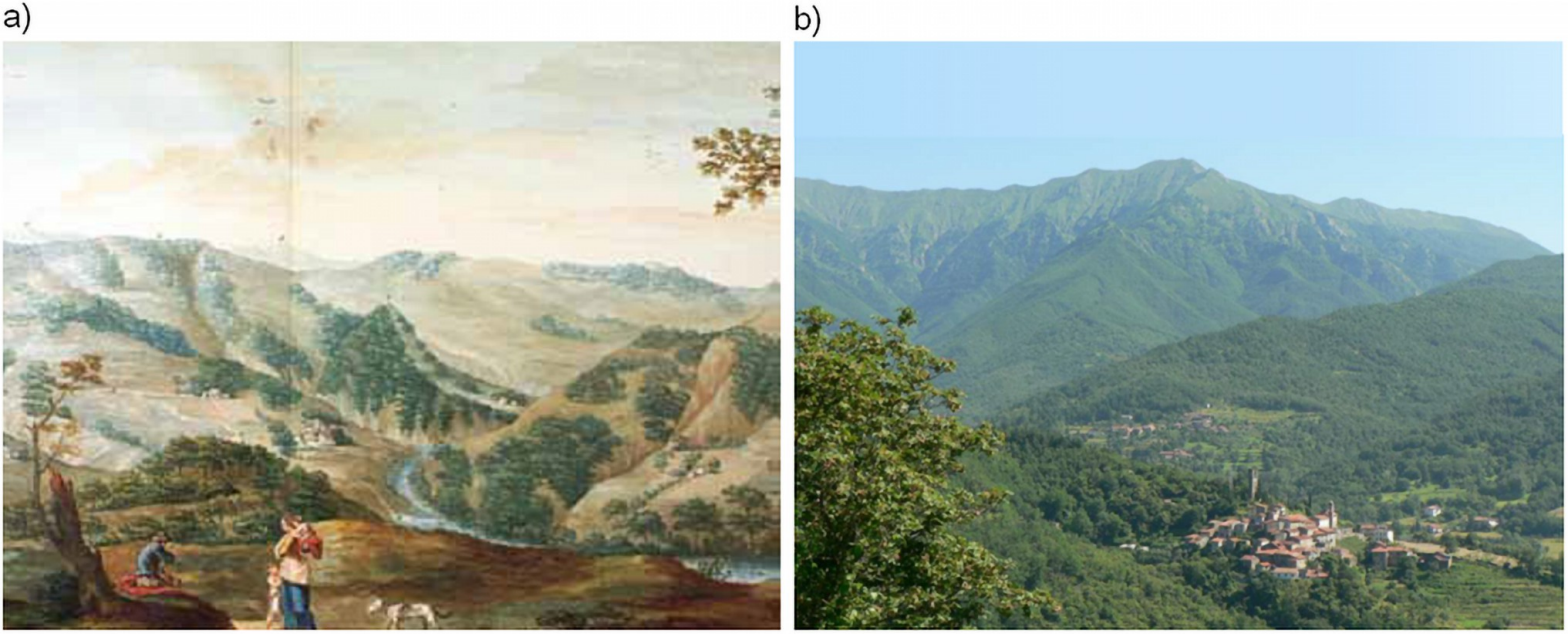
Environmental change along the history of Tuscany landscape. a) a view of upper Casentino with pastures and deforested mountains, *c*. 1780 by Pietro Ferroni (1745–1825) and collaborators [56]; State Archive of Florence, Italy, https://www.archiviodistato.firenze.it), b) Lunigiana mountainous landscape in a picture of Alberto Chiti-Batelli (born 1959), with medieval villages, terraced agricultural areas near the villages, chestnut woods, beech woods and summit meadows for summer grazing ([56]; source: Nature and Environment Management Operators, Florence, Italy, http://www.nemoambiente.com/galleria).

More recent studies [11, 33], on the other hand, refer to a historical phase of minornarrowing, observed from the late 19^th^ century and the 1950s, explained as a response to basin-scale disturbances through a slow process of abandonment and permanent migration by the smallholder farming: forest develoment (both coppice and tall trees) becoming the main economic resource, strong reduction of forest-dependent and uncultivated agricultural space and thus widespread renaturation, with extensive reforestation (especially of conifers), especially as a consequence of the 1923 forestry law [55] (Fig 5).

**Fig 6:**
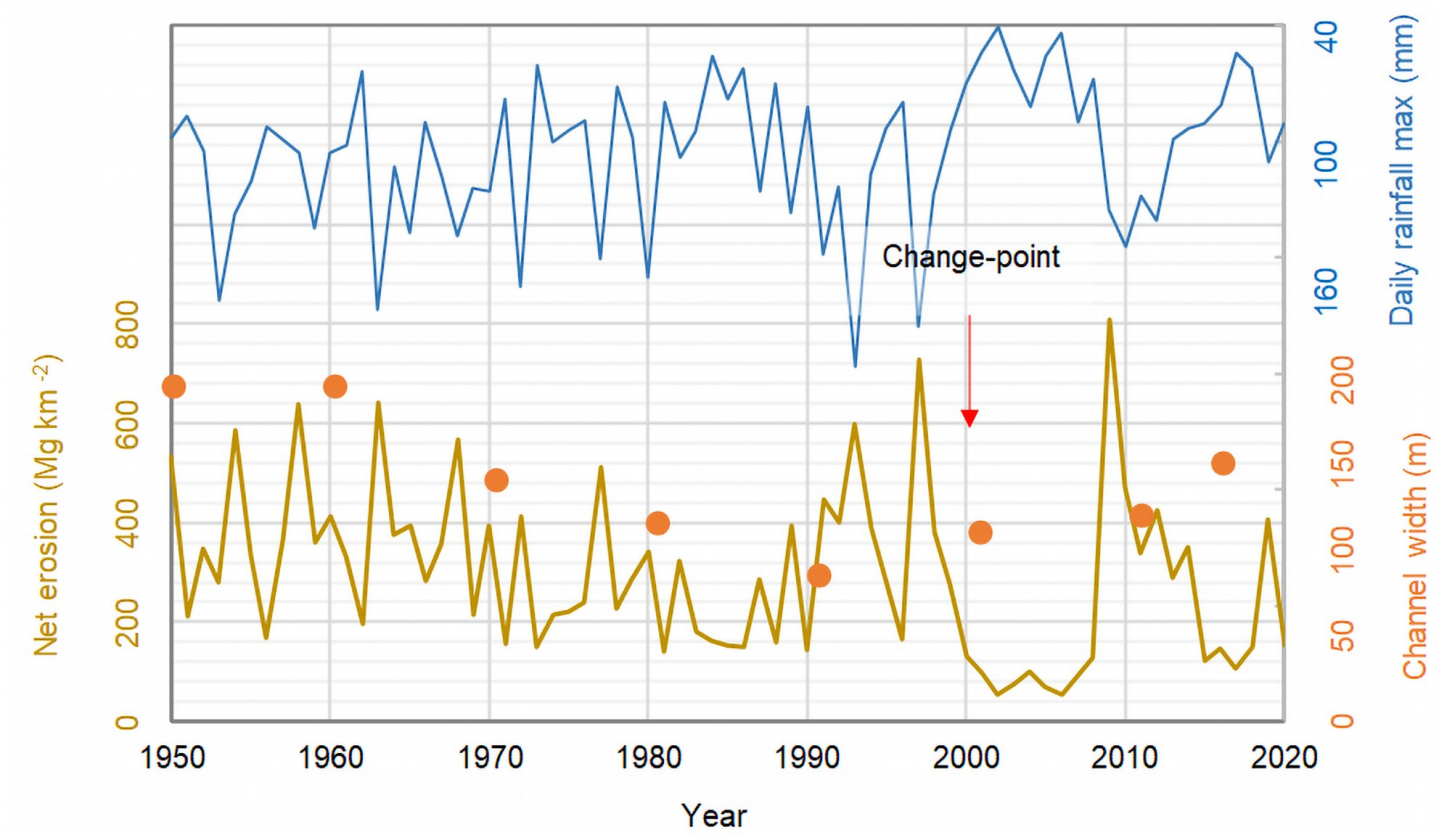
Evolution of annual net erosion for the Magra River Basin (ocher curve) during the period 1950–2020, as modelled by Eq (1), and overall pattern of changes in Magra channel width (orange dots; [27]). The annual maximum daily rainfall is also reported (blue curve).

With the recovery of the forests, both the chestnut groves deteriorated with a post-war crisis of the sharecropping system, despite the specialisation in zootechnics and forestry. This brings us to the years in which our model can work and give us a more objective and clearer evolution of sediment yield (Fig 6, ocher curve).

### Estimated time-series of annual net soil erosion

The REDEM_(MRB)_ estimates show over the 71-year reconstruction period (1950–2020) a mean value of 297 Mg km^-2^ yr^-1^ (±168 Mg km^-2^ yr^-1^ standard deviation). However, a change-point detected in the year 1999 by the SNHT-double shift [57] and Mann-Withney-Pettit [58] tests divides the time-series in two distinct time segments: 1950–1999 and 2000–2020 (Fig 6, red arrow). Rinaldi et al. [11] have also shown that bed incision was the predominant scenario during the time interval from the early 20^th^ century to the mid-1990s, while recent profiles indicate a following predominant phase of aggradation. According to the above change-point, the first period is affected by a moderate decline in sediment yield, with a mean and standard deviation of 330±149 Mg km^-2^ yr^-1^, compared to 218±190 Mg km^-2^ yr^-1^ in the second period. For both periods, the values of the terrigenous input at river mouths clearly reflect the characteristics of climate and vegetation cover, and secondarily of soil erodibility, highlighting in particular the considerable role in limiting sediment transport played by anthropogenic and natural landscape elements (Fig 2). They include agricultural abandonment that began in 1950 in *Val di Magra*, the renaturation and depopulation of the countryside with the loss or alteration of historical features (peasant houses, farming villages, barns and highland pastures), and reforestation [55]. The decrease in the sedimentation rate between 1950 and 1999 was also accompanied by a more contained interannual fluctuation, despite the intensive sediment mining, which occurred between the 1960s and 1980s [11]. This long-term decline in sediment yield is consistent with the decrease in the width of the Magra channel in the period 1951–1990 and the partial recovery after that date (Fig 6, orange dots; [27]). After 1980, the MRB experienced an increase in greenness, suggesting an enrichment of woodland and grassland, until the 2000s. This suggests that vegetation cover was not sufficient to prevent erosion of the banks of the sinuous reaches, but may have contributed to promoting the deposition of bed material on the floodplain by increasing roughness and favouring the tendency to avulsion [27]. However, this scattered recovery in the more recent phase 2000–2020 also has a more unstable evolutionary character. This part of the time-series is thus much more inconsistent and hazardous, although the mean value is lower. We see that the sedimentation rates undergo a notable oscillation around the critical value of tolerable soil loss, passing from one extreme to the other, with a large phase of quiet erosive sedimentation. Finally, this phase of partial recovery of channel width can be recognised and explained by a renewed sediment supply and mobility promoted by a series of flood events (especially the October 2011 flood) together with a delayed response to the cessation of intensive sediment exploitation [27].

This indicates that the regulation of soil and water conservation measures on the spatio-temporal scale effects of the rainfall-erosion process in the MRB is in an unstable equilibrium, where events close to moderate-extreme storms can, at times, determine non-tolerable conditions in soil loss. In this context, the year 2009 deserves a distinct mention, as it recorded the highest absolute sediment rate in the time-series (803 Mg km^-2^), due to the floods that affected northern Tuscany at the end of that year. The events of 23 and 25 December were caused by a series of hydrological criticalities scattered throughout the Lunigiana river network, both main and minor, essentially due to local fluvial dynamics (erosion, overflows) and failures of containment measures [59]. However, these hydro-geomorphological extremes do not correspond to a maximum peak of daily rainfall. The hypothesis is that simplification of the agricultural network in some sections of the Magra River valley floor (increase in the size of the plots, homogenisation of crops, dominance of arable crops, elimination of the accompanying vegetation and parts of the historical drainage network; [56]) could have favoured pulses in sediment production at the outlet of the basin. Moreover, since 2000, in some areas of the upper Magra River valley, such as the Filattiera Plain, considerable channel sedimentation from eroding slopes has started to prevail, linked to the dynamics of abandonment of settlement and rural systems in the hills and mountainous areas [56]. Previous studies [60, 61] have indicated that sediment supply from hillslopes was a key determinant of channel response during extreme flood events. Although these studies provide only a partial representation of the overall change in bed elevation, they are consistent with observations made during the post-flood field survey, when deposition of significant amounts of fresh sediment along the bars and adjacent floodplain was observed [27]. Aerial photographs taken before and after the flood of 25 October 2011 facilitate the geographical identification of the watercourses affected by the flood, in the perspective view of the upper Magra valley. The watercourses have widened their bed both upstream of the Magra valley) and downstream of the Filattiera Plain, where the Magra River itself has widened its bed by many meters, and where sediment deposits are evident on both sides of the river.

### Changes in climate and landscape patterns

Considering the reconstructed time-series examined so far in its evolution, it can be assumed that storms grafted onto a longer-term climate together with the vegetation cycle are an example of important variations in climatic and landscape patterns, as the SDR was affected by few variations both along the seasons and in the multi-year evolution. Analysis of several historical time-series of total annual precipitation at different stations in the MRB [36] did not reveal any significant trend in the 20^th^ century, with only a modest reduction in precipitation over time. Our modelling study separated the hydrological input from the maximum annual daily precipitation. The result is reported in Fig 6 (blue curves), where the Mann-Kendall test [62] returned a significant decrease (p = 0.03) of the latter in the period analysed. Thus, although the recent extreme storms associated with sediment yield are characterized by lower cumulative rainfall, they indicate a greater propensity of the basin to produce pulsed sediment over time. This is consistent with the increased frequency of flash floods during recent decades in the Mediterranean area [63–65].

On the other hand, the sediment runoff recorded during the last period, despite a continuous decrease in extreme precipitation, has stopped its descent, sometimes trigging remarkable pulses, as in 1993, 1997 and 2009. Vegetation cover also played an important role in the middle part of the time-series, when the reforestation of many areas made it possible to better contain sediment transport downstream, even during extreme events.

We cannot know how long this trend will remain unchanged, as there have been years with marked variability, but it is certain that under these conditions it becomes difficult to plan agricultural activities. However, land managers must always be aware of this trend, as it is almost impossible to know which year will have its extreme value with considerable losses of sediment, organic matter and soil nutrients.

### Basin-wide modelling of soil erosion

This research has taken a major step towards the modelling of soil erosion in a Mediterranean fluvial basin. The analysis conducted in the MRB between observed changes in sediment discharge and selected environmental controlling factors revealed that there are strong interrelationships that explain the temporal pattern and variability of erosion rate. The use of a parsimonious model has offered an interesting possibility to reconstruct net erosion time-series on a monthly basis. Although the model developed for the MRB may not be easily transferable for applications in other river basins, it provided a singular opportunity to model erosion responses to climate and land cover changes, where documented hydrological processes support the interpretation of the results. Through continuous observation of selected physical environmental variables, we were able to establish seasonal patterns of weathering processes and identify the factors that control erosivity and rainfall runoff and, in turn, net erosion. The main characteristic observed is the reaction of the MRB to precipitation events. Hydrological events show high fluctuations of suspended sediment from month to month, and from year to year, derived from a heterogeneous temporal distribution related to seasonal variations of hydroclimatic forcing (i.e. surface erosivity and runoff) and vegetation cover. We conclude that while sediment has always entered the Magra River mainly in discrete pulses associated with natural climatic oscillations, land abandonment and revegetation are the main causes of the observed reduction in net soil erosion in recent decades. This study adds to the body of literature on the development of methodological frameworks and tools that could be used to outline soil erosion and instability risk scenarios resulting from climatic changes (e.g. increased intense precipitation) and changes in land use and management in Mediterranean fluvial basins.

## Supporting information

S1 File (XLSX)

## References

[1] Benito G, Macklin MG, Panin A, Rossato S, Fontana A, Jones AF, et al. Recurring flood distribution patterns related to short-term Holocene climatic variability. Sci Rep 2015; 5:16398. doi: 10.1038/srep16398 26549043 PMC4637870

[2] Diodato N, Ljungqvist FC, Bellocchi G. Fingerprint of climate change in precipitation aggressiveness across the central Mediterranean (Italian) area. Sci Rep 2020; 10:22062. doi: 10.1038/s41598-020-78857-3 33328541 PMC7744579

[3] Diodato N, Ljungqvist FC, Bellocchi G. Climate patterns in the world’s longest history of storm-erosivity: the Arno River Basin, Italy, 1000–2019 CE. Front Earth Sci 2021; 9:637973.

[4] Zhang J, Shang Y, Liu J, Fu J, Wei S, Tong L. Causes of variations in sediment yield in the Jinghe River Basin, China. Sci Rep 2020; 10:18054. doi: 10.1038/s41598-020-74980-3 33093547 PMC7581752

[5] Thomas MF. Landscape sensitivity in time and space—An introduction. Catena 2001; 42:83–98.

[6] Caporali E, Lompi M, Pacetti T, Chiarello V, Fatichi S. A review of studies on observed precipitation trends in Italy. Int J Clim 2021; 41:E1–E25

[7] Ricci F Taglio del bosco, dilavamento delle acque e inondazioni nel bacino dell’Arno durante la seconda metà del Cinquecento. In: Bianca C, Salvestrini F, editors. L’acqua nemica. Fiumi, inondazioni e città storiche dall’antichità al contemporaneo. Spoleto: Fondazione CISAM; 2017. p. 205–39. Italian

[8] Sereni E Storia del paesaggio agrario Italiano. Bari: Laterza; 1961. Italian

[9] Rinaldi M, Simon A. Bed-level adjustments in the Arno River, Central Italy. Geomorphology 1998; 22:57–71.

[10] Rinaldi M Recent channel adjustments in alluvial rivers of Tuscany, Central Italy. Earth Surf Process Landf 2003; 28:587–608.

[11] Rinaldi M, Simoncini C, Piégay H. Scientific design strategy for promoting sustainable sediment management: the case of the Magra River (Central‐Northern Italy). River Res Appl 2009; 25:607–25.

[12] De Vente J, Poesen J, Verstraeten G, Govers G, Vanmaercke M, Van Rompaey A, et al. Predicting soil erosion and sediment yield at regional scales: Where do we stand? Earth Sci Rev 2013; 127:16–29.

[13] Jakeman AJ, Green TR, Beavis SG, Zhang L, Dietrich CR, Crapper PF Modelling upland and instream erosion, sediment and phosphorus transport in a large catchment. Hydrol Process 1999; 13:745–52.

[14] Mouyen M, Longuevergne L, Steer P, Crave A, Lemoine J-M, Save H, et al. Assessing modern river sediment discharge to the ocean using satellite gravimetry. Nat Commun 2018; 9:3384. doi: 10.1038/s41467-018-05921-y 30139937 PMC6107634

[15] Brazier R. Hillslope soil erosion modeling. In: Shroder JF, editor. Treatise on geomorphology. San Diego: Elsevier; 2013. vol. 2, p. 135–46.

[16] Merrit W, Letcher RA, Jakeman AJ. A review of erosion and sediment transport models. Environ Model Softw 2003; 18:761–99.

[17] Noe GB, Cashman MJ, Skalak K, Gellis A, Hopkins KG, Moyer D, et al. Sediment dynamics and implications for management: State of the science from long-term research in the Chesapeake Bay watershed, USA. WIREs Water 2020; 7:e1454.

[18] De Vente J, Poesen J, Verstraeten G The application of semi-quantitative methods and reservoir sedimentation rates for the prediction of basin sediment yield in Spain. J Hydrol 2005; 305:63–86.

[19] Corella JP, Valero-Garcés PL, Vicente-Serrano SM, Brauer A, Benito G. Three millennia of heavy rainfalls in Western Mediterranean: frequency, seasonality and atmospheric drivers. Sci Rep 2016; 6:1–11. 27910953 10.1038/srep38206 PMC5133600

[20] Faccini F, Luino F, Paliaga G, Sacchini A, Turconi L, Jong C Role of rainfall intensity and urban sprawl in the 2014 flash flood in Genoa City, Bisagno catchment (Liguria, Italy). Appl Geogr 2018; 98:224–41.

[21] Brandolini P, Cevasco A, Capolongo D, Pepe G, Lovergine F, Del Monte M Response of terraced slopes to a very intense rainfall event and relationships with land abandonment: A case study from Cinque Terre (Italy). Land Degrad Dev 2018; 29:630–42.

[22] Cevasco A, Diodato N, Revellino P, Fiorillo F, Grelle G, Guadagno FM. Storminess and geo-hydrological events affecting small coastal basins in a terraced Mediterranean environment. Sci Total Environ 2015; 532:208–19. doi: 10.1016/j.scitotenv.2015.06.017 26071962

[23] Lucía A, Comiti F, Borga M, Cavalli M, Marchi L Dynamics of large wood during a flash flood in two mountain catchments. Nat Hazards Earth Syst Sci Discuss 2015; 3:1643–80.

[24] Amponsah W, Marchi L, Zoccatelli D, Boni G, Cavalli M, Comiti F., et al. Hydrometeorological characterization of a flash flood associated with major geomorphic effects: Assessment of peak discharge uncertainties and analysis of the runoff response. J Hydrometeorol 2016; 17:3063–77.

[25] Rinaldi M, Belletti B, Bussettini M, Comiti F, Golfieri B, Lastoria B, et al. New tools for the hydromorphological assessment and monitoring of European streams. J Environ Manage 2016; 202:363–78. doi: 10.1016/j.jenvman.2016.11.036 27889363

[26] Bartelletti C, Giannecchini R, D’Amato Avanzi G, Galanti Y, Mazzali A. The influence of geological–morphological and land use settings on shallow landslides in the Pogliaschina T. basin (northern Apennines, Italy). J Maps 2017; 13:142–52.

[27] Nardi L, Rinaldi M. Spatio-temporal patterns of channel changes in response to a major flood event: the case of the Magra River (central–northern Italy). Earth Surf Process Landf 2015; 40:326–39.

[28] Prosdocimi M, Cerdà A, Tarolli P Soil water erosion on Mediterranean vineyards: A review. Catena 2016; 141:1–21.

[29] Foster GR, Meyer LD, Onstad CA A runoff erosivity factor and variable slope length exponents for soil loss estimates. Trans ASAE 1977; 20:683–687.

[30] Thornes JB The interaction of erosional and vegetational dynamics in land degradation: Spatial outcomes. In: Thornes JB, editor. Vegetation and erosion: Processes and environments. Chichester: John Wiley & Sons; 1990. pp. 45–55.

[31] Ballabio C, Borrelli P, Spinoni J, Meusburger K, Michaelides S, Beguería S, et al. Mapping monthly rainfall erosivity in Europe. Sci Total Environ 2017; 579:1298–1315. doi: 10.1016/j.scitotenv.2016.11.123 27913025 PMC5206222

[32] Rinaldi M, Simoncini C. Studio geomorfologico del Fiume Magra e del Fiume Vara finalizzato alla gestione dei sedimenti e della fascia di mobilità. In: Nuovi approcci per la comprensione dei processi fluviali e la gestione dei sedimenti. Applicazioni nel bacino del Magra. Sarzana: Magra River Basin Authority; 2006. pp. 93–109.

[33] Rinaldi M, Teruggi LB, Simoncini C, Nardi L. Dinamica recente ed attuale di alvai fluviali: alcuni casi di studio dell’Appennino settentrionale. Il Quaternario—Italian Journal of Quaternary Sciences 2008; 21:291–302.

[34] Panizza M. Geomorfologia. Bologna: Pitagora; 1992. Italian

[35] Rinaldi M. Studio geomorfologico dei principali alvei fluviali nel bacino del fiume Magra finalizzato alla definizione di linee guida di gestione dei sedimenti e della fascia di mobilità funzionale. Florence: Autorità di Bacino del Fiume Magra, Department of Civil Engineering—University of Florence; 2005. Italian

[36] Alaimo A, Aru S, Donadelli G, Nebbia F. Geografie di oggi. Metodi e strategie tra ricerca e didattica. Milan: Franco Angeli; 2015. Italian

[37] Rapetti F, Vittorini S. I caratteri del clima. In: Mazzanti R., editor. Tirrenia: Edizioni del Cerro; 1994. p. 103–31.

[38] Walter H, Lieth HHF. Klimadiagramm-Weltatlas. Jena: G. Fischer Verlag; 1967. German

[39] Diodato N. Predicting RUSLE (Revised Universal Soil Loss Equation) monthly erosivity index from readily available rainfall data in Mediterranean area. The Environmentalist 2005; 25:63–70.

[40] Vermote EF. MOD09A1 v006. Improvements/changes from previous versions. https://lpdaac.usgs.gov/products/mod09a1v006; 2019.

[41] Toy TJ, Foster GR, Renard KG. Soil erosion; prediction, measurement, and control. New York: John Wiley & Sons; 2002.

[42] Diodato N, Aronica G. Finding simplicity in storm erosivity modelling. In: Diodato N, Bellocchi G, editors. Storminess and environmental change: climate forcing and response in the Mediterranean region. Dordrecht: Springer; 2014. pp. 53–64.

[43] Shojaei S, Kalantari Z, Rodrigo-Comino J. Prediction of factors affecting activation of soil erosion by mathematical modeling at pedon scale under laboratory conditions. Sci Rep 2020; 10:20163. doi: 10.1038/s41598-020-76926-1 33214590 PMC7677555

[44] Zhang J, Zhang X, Li R, Chen L, Lin P. Did streamflow or suspended sediment concentration changes reduce sediment load in the middle reaches of the Yellow River? J Hydrol 2017; 546:357–69.

[45] Wu L, Peng W, Qiao S, Ma X. Effects of rainfall intensity and slope gradient on runoff and sediment yield characteristics of bare loess soil. Environ Sci Pollut Res 2018; 25:3480–87. doi: 10.1007/s11356-017-0713-8 29159433

[46] Arnold J, Williams J, Maidment DR. Continuous-time water and sediment routing model for large basins. J Hydraul Eng 1995; 121:171–83.

[47] Quinton JN, Catt J.A, Wood GA, Steer J. Soil carbon losses by water erosion: Experimentation and modeling at field and national scales in the UK. Agric Ecosyst Environ 2006; 112:87–102.

[48] Royston P, Sauerbrei W. Multivariate model–building: A pragmatic approach to regression anaylsis based on fractional polynomials for modelling continuous variables. New Yoork: John Wiley & Sons; 2008.

[49] Huss M, Farinotti D, Bauder A. Modelling runoff from highly glacierised alpine drainage basins in a changing climate. Hydrol Process 2008; 22:3888–902.

[50] Rainato R, Picco L, Cavalli M, Mao L, Neverman AJ, Tarolli P. Coupling climate conditions, sediment sources and sediment transport in an Alpine basin. Land Degrad Dev 2017; 29:1154–66.

[51] Mulligan M, Wainwright J. Modelling and model building. In: Wainwright J, Mulligan M, editors. Environmental modelling: finding simplicity in complexity. West Sussex: John Wiley & Sons Ltd; 2004. pp. 7–73.

[52] Borselli L, Cassi P, Sanchis PS, Ungaro F. Studio della dinamica delle aree sorgenti primarie di sedimento nell’area pilota del Bacino di Bilancino: Progetto BABI. Florence: Consiglio Nazionale delle Ricerche—Istituto di Ricerca per la Protezione Idrogeologica (CNR-IRPI)–Unità “Pedologia Applicata”; 2004. Italian

[53] Surian N, Righini M, Lucía A, Nardi L, Amponsah W, Benvenuti M, et al. Channel response to extreme floods: Insights on controlling factors from six mountain rivers in northern Apennines, Italy. Geomorphology 2016; 272:78–91.

[54] Targioni Tozzetti G. Relazioni d’alcuni viaggi fatti in diverse parti della Toscana, XI. Florence: Florence: Stamperia granducale; 1777.

[55] Regione Toscana. Lunigiana, Piano di Indirizzo Territoriale con valenza di piano paesaggistico; 2015. Italian

[56] Regione Toscana. Piano I paesaggi rurali storici della Toscana, Piano di Indirizzo Territoriale con valenza di piano paesaggistico; 2012. Italian

[57] Alexandersson H, Moberg A. Homogenization of Swedish temperature data. Part I: homogeneity test for linear trends. Int J Climatol 1997; 17:25–34.

[58] Pettitt AN. A non-parametric approach to the change-point problem. Appl Stat 1979; 28:126–35.

[59] SIR. Report sull’evento alluvionale registrato nei giorni 24–25 dicembre 2009 nel Bacino del Fiume Serchio. Florence: Regione Toscana–Servizio Idrologico Regionale Centro Funzionale della Regione Toscana; 2010. Italian

[60] Harvey AM. Coupling between hillslopes and channels in upland fluvial systems: implications for landscape sensitivity, illustrated from the Howgill Fells, northwest England. Catena 2001; 42:225–50.

[61] Sloan J, Miller JR, Lancaster N Response and recovery of the Eel River, California, and its tributaries to floods in 1955, 1964, and 1997. Geomorphology 2001; 36:129–54.

[62] Kendall MG Rank correlation methods, 4th ed. London: Charles Griffin; 1975.

[63] Llasat MC, Llasat-Botija M, Prat MA, Porcu F, Price C, Mugnai A, et al. High-impact floods and flash floods in Mediterranean countries: the FLASH preliminary database. Adv Geosci 2010; 23:47–55.

[64] Tarolli P, Borga M, Morin E, Delrieu G. Analysis of flash flood regimes in the north-western and south-eastern Mediterranean regions. Nat Hazards Earth Syst Sci 2012; 12:1255–65.

[65] Flaounas E, Drobinski P, Vrac M, Bastin S, Lebeaupin-Brossier C, Stéfanon M, et al. Precipitation and temperature space–time variability and extremes in the Mediterranean region: evaluation of dynamical and statistical downscaling methods. Clim Dyn 2013; 40:2687–705.

